# The Myrmecofauna (Hymenoptera: Formicidae) of Hungary: Survey of Ant Species with an Annotated Synonymic Inventory

**DOI:** 10.3390/insects12010078

**Published:** 2021-01-16

**Authors:** Sándor Csősz, Ferenc Báthori, László Gallé, Gábor Lőrinczi, István Maák, András Tartally, Éva Kovács, Anna Ágnes Somogyi, Bálint Markó

**Affiliations:** 1 MTA-ELTE-MTM Ecology Research Group, Pázmány Péter sétány 1/C, 1117 Budapest, Hungary; sandorcsosz2@gmail.com; 2 Evolutionary Ecology Research Group, Centre for Ecological Research, Institute of Ecology and Botany, 2163 Vácrátót, Hungary; ferenc.bathori@gmail.com; 3 Department of Ecology and Natural History Collection, University of Szeged, Szeged Boldogasszony sgt. 17., 6722 Szeged, Hungary; galle@bio.u-szeged.hu; 4 Department of Ecology, University of Szeged, Közép fasor 52, 6726 Szeged, Hungary; lorinczig@gmail.com (G.L.); bikmakk@gmail.com (I.M.); 5 Museum and Institute of Zoology, Polish Academy of Sciences, ul. Wilcza 64, 00-679 Warsaw, Poland; 6 Department of Evolutionary Zoology and Human Biology, University of Debrecen, Egyetem tér 1, 4032 Debrecen, Hungary; panka.somogyi@gmail.com; 7 Kiskunság National Park Directorate, Liszt F. u. 19, 6000 Kecskemét, Hungary; kovacse@knp.hu; 8 Hungarian Department of Biology and Ecology, Babeş-Bolyai University, Clinicilor 5-7, 400006 Cluj-Napoca, Romania; balintm@gmail.com; 9 Centre for Systems Biology, Biodiversity and Bioresources, Babeș-Bolyai University, Clinicilor 5-7, 400006 Cluj-Napoca, Romania

**Keywords:** ants, biogeography, faunistics, checklist, Europe

## Abstract

**Simple Summary:**

Abundance is a hallmark of ants (Hymenoptera: Formicidae). They are exceedingly common in both natural and artificial environments and they constitute a conspicuous part of the terrestrial ecosystem; every 3 to 4 out of 10 kg of insects are given by ants. Due to their key role in natural habitats, they are at the basis of any nature conservation and pest management policy. Thus, the first step in developing adequate management strategies is to build a precise faunistic inventory. More than 16,000 valid ant species are registered worldwide, of which 126 are known to occur in Hungary. Thanks to the last decade’s efforts in the Hungarian myrmecological research, and because of the constantly changing taxonomy of several problematic ant genera, a new checklist of the Hungarian ants is presented here. A comparison of the Hungarian myrmecofauna to other European countries’ ant fauna is also provided in this paper. The current dataset is a result of ongoing work on inventorying the Hungarian ant fauna, therefore it is expected to change over time and will be updated once the ongoing taxonomic projects are completed.

**Abstract:**

Ants (Hymenoptera: Forimicidae) are exceedingly common in nature. They constitute a conspicuous part of the terrestrial animal biomass and are also considered common ecosystem engineers. Due to their key role in natural habitats, they are at the basis of any nature conservation policy. Thus, the first step in developing adequate conservation and management policies is to build a precise faunistic inventory. More than 16,000 valid ant species are registered worldwide, of which 126 are known to occur in Hungary. Thanks to the last decade’s efforts in the Hungarian myrmecological research, and because of the constantly changing taxonomy of several problematic ant genera, a new checklist of the Hungarian ants is presented here. The state of the Hungarian myrmecofauna is also discussed in the context of other European countries’ ant fauna. Six species (*Formica lemani*, *Lasius nitidigaster*, *Tetramorium immigrans*, *T. staerckei*, *T. indocile* and *Temnothorax turcicus*) have been reported for the first time in the Hungarian literature, nine taxon names were changed after systematic replacements, nomenclatorial act, or as a result of splitting formerly considered continuous populations into more taxa. Two species formerly believed to occur in Hungary are now excluded from the updated list. All names are nomenclaturally assessed, and complete synonymies applied in the Hungarian literature for a certain taxon are provided. Wherever it is not self-evident, comments are added, especially to explain replacements of taxon names. Finally, we present a brief descriptive comparison of the Hungarian myrmecofauna with the ant fauna of the surrounding countries. The current dataset is a result of ongoing work on inventorying the Hungarian ant fauna, therefore it is expected to change over time and will be updated once the ongoing taxonomic projects are completed.

## 1. Introduction

Faunistic papers and regional checklists are cornerstones of nature conservation. Without up-to-date faunistic information, it is almost impossible to formulate adequate conservation strategies for species or specific habitats [1,2,3]. As a drastic decline in species number in the past decades is widely acknowledged [4,5], public interest for biodiversity is constantly growing, and there is an increasing demand for accurate faunistic information also from non-academics. This is proven by the existence of numerous specific interest groups on social media bringing together specialists and amateurs alike. Citizen science, the implication of volunteering non-academics in scientific research, could be essential for inventorying biota [6], saving habitats, protecting species [7,8], describing new taxa [9], and detecting new fauna elements [10,11]. It could also help overcome to a certain degree the ongoing crisis of taxonomic impediment, namely the lack of specialists [12]. Thus, a periodical update of any checklist should be considered as almost mandatory for assisting conservation policies. In the frame of the current study, we offer an updated version of the Hungarian ant fauna with necessary corrections and also additions due to novel findings in the past years.

The myrmecofauna of central and eastern Europe is relatively well-known as over the course of the past few decades several new checklists, faunistic monographs and keys have been published for this region and generally for Europe [13,14,15,16,17,18,19,20,21,22,23]. The last checklist of Hungarian ants was compiled in 2011 and reported 126 species [24].

The myrmecofauna of present-day Hungary has been studied in considerable detail over the course of the last few centuries, and several checklists have been published since the mid-1800s. The first detailed checklist containing locations was published by Mayr [25]. He listed 40 species for the territory of present-day Hungary. His work was later updated toward the end of the 19th century [26], resulting in a checklist that contained 56 species for the territory of present-day Hungary. More than 50 years later the monograph of Somfai [27] listed still just 66 species, which could be considered quite low for this region even by the standards of that time. The first checklist that, indeed, passed the threshold of 100 species, was published in Gallé et al. [28] that already contained 101 species, and also resolved many taxonomic and faunistic inconsistencies. The number of species increased considerably in the last checklist of Hungarian ants published in Csősz et al. [24], containing 122 ant species, mostly due to the increase in the number of myrmecologist scholars and also their faunistic and taxonomical works [29].

Despite this improvement over the last few decades, since the publication of the latest Hungarian checklist, many new taxa have been described [30,31], revived from synonymy [32], or the validity of their status has been confirmed or even dismissed on the basis of new findings [15,23]. Furthermore, several new myrmecological studies have been carried out reporting new ant species for the Hungarian fauna and new data regarding species already known [29,33]. Consequently, there is a need for an updated checklist of the Hungarian myrmecofauna, based on the most up to date taxonomic knowledge.

## 2. Materials and Methods

The current list of species was prepared by using every major faunistic paper concerning the territory of current Hungary, as well as other papers handling Hungarian data. For the up-to-date nomenclature, taxonomy, and systematics of ants, we followed Bolton’s Catalogue and Synopsis [34]. Most species were collected over the course of the recent decade, but there are some records that could not be verified, neither by checking voucher specimens nor by collecting from the sample sites.

## 3. Results

The total number of species is 126 belonging to 33 genera, out of which six are new elements for the Hungarian fauna: *Formica lemani*, *Lasius nitidigaster*, *Tetramorium immigrans*, *T. staerckei*, *T. indocile* and *Temnothorax turcicus* have been identified after the last check-list [24]. Five species of the exotic origin occur exclusively indoors: *Hypoponera punctatissima*, *Monomorium pharaonis*, *Tetramorium bicarinatum*, *T. insolens* and *Tapinoma melanocephalum*. Two species, *Temnothorax rabaudi* and *Tetramorium impurum*, are considered to be of uncertain occurrence, hence these species are now excluded from the Hungarian myrmecofauna, as voucher specimens or recent investigations do not support their presence.

### 3.1. Subfamily: Proceratiinae

#### 3.1.1. Tribe: Proceratiini

##### Genus: *Proceratium*

*P. melinum* (Roger, 1860)

        = *Sysphincta fialai* Kratochvil, 1944: [27]

### 3.2. Subfamily: Ponerinae

#### 3.2.1. Tribe: Ponerini

##### Genus: *Cryptopone*

*C. ochracea* (Mayr, 1855)

        = *Cryptopone ochraceum*: [35]

##### Genus: *Hypoponera*

*H. punctatissima* (Roger, 1859): [28] (Almost exclusively an indoor tramp species)

        = *Ponera punctatissima*: [27]

##### Genus: *Ponera*

*P. coarctata* (Latreille, 1802): [25]

*P. testacea*Emery, 1895: [36]

### 3.3. Subfamily: Myrmicinae

#### 3.3.1. Tribe: Attini

##### Genus: *Strumigenys*

*S. argiola* (Emery, 1869)

        = *Pyramica argiola*: [24]

        = *Epitritus argiolus*: [26]

*S. baudueri* (Emery, 1875)

        = *Pyramica baudueri*: [24]

        = *Strumigenys baudueri*: [27]

        = *Smithistruma baudueri*: [28]

#### 3.3.2. Tribe: Crematogastrini

##### Genus: *Cardiocondyla*

*C. dalmatica*Soudek, 1925: [23]

        = *Cardiocondyla sahlbergi* Forel, 1913: [28] (erroneous determination)

        = *Cardiocondyla elegans* Emery, 1869: [24] (erroneous determination)

##### Genus: *Crematogaster*

*C. schmidti* (Mayr, 1853): [28]

*C. scutellaris* (Olivier, 1792): [37]

*C. sordidula* (Nylander, 1849): [37]

##### Genus: *Formicoxenus*

*F. nitidulus* (Nylander, 1846): [27]

##### Genus: *Harpagoxenus*

*H. sublaevis* (Nylander, 1849): [27]

##### Genus: *Leptothorax*

*L. acervorum* (Fabricius, 1783): [27]

*L. gredleri*Mayr, 1855: [27]

*L. muscorum* (Nylander, 1846): [27]

##### Genus: *Myrmecina*

*M. graminicola* (Latreille, 1802): [27]

        = *Myrmecina latreillei* Curtis, 1829: [25]

##### Genus: *Strongylognathus*

*S. testaceus* (Schenck, 1852): [25]

##### Genus: *Temnothorax*

*T. affinis* (Mayr, 1855)

        = *Leptothorax affinis* Mayr, 1855: [27]

*T. albipennis* (Curtis, 1854): [24]

*T. clypeatus* (Mayr, 1853)

        = *Leptothorax clypeatus*: [27]

*T. corticalis* (Schenck, 1852)

        = *Leptothorax corticalis*: [27]

*T. crassispinus* (Karavajev, 1926)

        = *Leptothorax nylanderi*: [25]

        = *Leptothorax slavonicus* Seifert, 1995: [38]

*T. interruptus* (Schenck, 1852)

        = *Leptothorax interruptus* Mayr, 1855: [26]

        = *Leptothorax tuberum* var. *interruptus* André, 1881: [27]

*T. jailensis* (Arnoldi, 1977): [24]

*T. nigriceps* (Mayr, 1855)

        = *Leptothorax nigriceps* Mayr, 1855: [39]

*T. parvulus* (Schenck, 1852)

        = *Leptothorax parvulus* (Schenck, 1852): [27]

        *T. sordidulus* (Müller, 1923)

        = *Leptothorax sordidulus*: [39]

*T. tuberum* (Fabricius, 1775)

        = *Leptothorax tuberum* Mayr, 1855: [37]

*T. turcicus* (Santschi, 1934): new record, Iszkaszentgyörgy, 47.236382 N, 18.284685 E, 187 m, leg. S. Csősz, 06.06.2020 (3 workers), Budapest, Rupp-hegy, 47.4730 N, 18.9794 E, 197 m, leg. S. Csősz, 04.05.2018 (6 workers, 2 gynes), Budapest, Rupp-hegy, 47.4730 N, 18.9794 E, 197 m, leg. S. Csősz, 17.06.2019 (3 workers), Mátraháza, 47.850518 N, 19.959551 E, 584 m, leg. S. Csősz, 04.08.2019 (3 workers)

*T. unifasciatus* (Latreille, 1798): [40]

        = *Leptothorax unifasciatus*: [25]

        = *Leptothorax tuberum* var. *unifasciata* André, 1881: [40]

*T. zaleskyi* (Sadil 1953)

        = *Myrmoxenus ravouxi* (André, 1896)

        = *Epimyrma goesswaldi* Menozzi, 1930: [41]

        = *Epimyrma ravouxi*: [38]

##### Genus: *Tetramorium*

*T. atratulum*: [25]

        = *Anergates atratulus* (Schenck, 1952): [26]

*T. bicarinatum* (Nylander, 1846): [26] (Indoor tramp species)

*T. caespitum* (Linnaeus, 1758): [42]

*T. ferox*Ruzsky, 1903: [43]

*T. hungaricum*Röszler, 1935

        = *Tetramorium caespitum hungaricum* Röszler, 1935: [44]

        = *Tetramorium hungaricum*: [45,46]

*T. immigrans* Santschi, 1927

        = *Tetramorium* sp. E: [24,47]

*T. indocile* Santschi 1927: [48]

        = *Tetramorium* sp. C: [24,47]

*T. insolens* (Smith, 1861): [27] (Indoor tramp species)

*T. moravicum*Kratochvíl, 1941

        = *Tetramorium rhenanum* Schulz 1996: [28]

        = *Tetramorium moravicum*: [49]

*T. semilaeve* (André, 1883): [28]

        = *Tetramorium simillimum* (Smith, 1851): [28] (erroneous determination)

*T. staerckei*Kratochvíl, 1944

        = *Tetramorium* sp. D: [24,47]

#### 3.3.3. Tribe: Myrmicini

##### Genus: *Manica*

*M. rubida* (Latreille, 1802): [28]

        = *Myrmica rubida* (Latreille, 1802): [42]

        = *Myrmica* (*Neomyrma*) *rubida*: [27]

##### Genus: *Myrmica*

*M. constricta*Karavajev, 1934: based on findings of Seifert et al. [50], *M. constricta* is an eastern European congener of *M. hellenica* that has long been considered to occur in Hungary. The formerly believed continuous population of “*M. hellenica*” is therefore split into two geographically distinct species and the Hungarian population belongs to *M. constricta*.

        = *Myrmica hellenica* Finzi, 1926: [51]

*M. curvithorax*Bondroit, 1920

        = *Myrmica slovaca* Sadil, 1952: [24]

        = *Myrmica salina* Ruzsky, 1905: [38]

*M. deplanata*Ruzsky, 1905: [27]

*M. gallienii*Bondroit, 1920: [38]

*M. karavajevi* (Arnoldi, 1930)

        = *Sifolinia karavajevi*: [28]

        = *Sifolinia faniensis* (van Boven, 1970): [52]

*M. lobicornis*Nylander, 1846: [26]

*M. lonae*Finzi, 1926: [53]

*M. rubra* (Linnaeus, 1758): [38]

        = *Myrmica laevinodis* Nylander, 1846: [25]

        = *Myrmica microrubra* Seifert, 1993: [54]

*M. ruginodis*Nylander, 1846: [42]

*M. rugulosa*Nylander, 1849: [26]

*M. sabuleti*Meinert, 1861: [27]

*M. scabrinodis*Nylander, 1846: [42]

        = *Myrmica rugulosoides* Forel, 1915: [55]

*M. schencki*Viereck, 1903: [56]

*M. specioides*Bondroit, 1918: [57]

        = *Myrmica sancta* Karavaiev, 1926: [58]

*M. vandeli*Bondroit, 1920: [53]

#### 3.3.4. Tribe: Solenopsidini

##### Genus: *Monomorium*

*M. pharaonis* (Linnaeus, 1758): [27] (Indoor tramp species)

##### Genus: *Solenopsis*

*S. fugax* (Latreille, 1798): [25]

#### 3.3.5. Tribe: Stenammini

##### Genus: *Aphaenogaster*

*A. subterranea* (Latreille, 1798): [26]

##### Genus: *Messor*

*M. structor* (Latreille, 1798): [27]

        = *Atta structor*: [42]

        = *Aphaenogaster structor*: [26]

        = *Messor rufitarsus* (Fabricius 1804): [59]

##### Genus: *Stenamma*

*S. debile* (Förster, 1850): [60]

        = *Stenamma westwoodii* Westwood, 1839: [27]

### 3.4. Subfamily: Dolichoderinae

#### 3.4.1. Tribe: Bothriomyrmecini

##### Genus: *Bothriomyrmex*

*B. communista* Santschi, 1919

        = *Bothriomyrmex meridionalis* (Roger, 1863): [27]

*B. corsicus* Santschi, 1923

        = *Bothriomyrmex menozzii* Emery, 1925: [61]

#### 3.4.2. Tribe: Dolichoderini

##### Genus: *Dolichoderus*

*D. quadripunctatus* (Linnaeus, 1771): [26]

        = *Hypoclinea quadripunctata*: [42]

#### 3.4.3. Tribe: Tapinomini

##### Genus: *Liometopum*

*L. microcephalum* (Panzer, 1798): [26]

        = *Formica austriaca* Mayr, 1853: [42]

##### Genus: *Tapinoma*

*T. erraticum* (Latreille, 1798): [42]

*T. melanocephalum* (Fabricius, 1793): [24] (Indoor tramp species)

*T. subboreale* Seifert, 2012: [62]

        = *Tapinoma madeirense* Forel, 1895: [24]

        = *Tapinoma ambiguum* Emery, 1925: [56]

### 3.5. Subfamily: Formicinae

#### 3.5.1. Tribe: Camponotini

##### Genus: *Camponotus*

*C. aethiops* (Latreille, 1798):

        = *Formica aethiops* Latreille, 1798: [42]

        = *Camponotus marginatus* Latreille, 1798: [26]

*C. atricolor* (Nylander, 1849) (sensu Seifert, 1996): [63]

*C. fallax* (Nylander, 1856): [64]

        = *Camponotus caryae* var. *fallax* (Nylander, 1856): [27]

        = *Camponotus caryae* (Fitch, 1855): [65]

*C. herculeanus* (Linnaeus, 1758): [26]

*C. lateralis* (Olivier, 1792): [26]

        = *Formica lateralis* Olivier, 1792: [42]

*C. ligniperda* (Latreille, 1802): [26]

*C. piceus* (Leach, 1825): [52]

        = *Camponotus lateralis* var. *piceus* (Leach, 1825): [27]

*C. tergestinus*Müller, 1921: [66]

*C. vagus* (Scopoli, 1763): [26]

        = *Formica pubescens* Fabricius, 1775: [25]

        = *Formica ligniperda*: [42]

##### Genus: *Colobopsis*

*C. truncata* (Spinola, 1808): [26]

#### 3.5.2. Tribe: Formicini

##### Genus: *Cataglyphis*

*C. aenescens* (Nylander, 1849): [28]

        = *Formica cursor* Fonscolombe, 1846: [25]

        = *Myrmecocystus cursor*: [26]

        = *Cataglyphis cursor aenescens*: [67]

*C. nodus* (Brullé, 1832): [28]

        = *Monocombus viaticus* (Fabricius, 1787): [42]

        = *Formica viatica*: [25]

        = *Cataglyphis viaticus* var. *orientalis* Forel, 1895: [26]

        = *Myrmecocystus bicolor* (Fabricius, 1793): [27]

        = *Cataglyphis bicolor nodus* (Brullé, 1832): [52]

##### Genus: *Formica*

*F. cinerea*Mayr, 1853: [27]

*F. clara* Forel, 1886

        = *Formica glauca* Ruzsky, 1895: [51]

        = *Formica lusatica* (Seifert 1997): [68]

*F. cunicularia*Latreille, 1798: [25]

        = *Formica fusca glebaria* Nylander, 1846: [67]

*F. exsecta*Nylander, 1846: [25]

*F. fusca*Linnaeus, 1758: [25]

*F. fuscocinerea*Forel, 1874: [54]

*F. gagates*Latreille, 1798: [42]

*F. lemani*Bondroit, 1917: new record, Veszprém m. Szigliget, 20-22.04.2019, leg. Z. Vas (3 workers)

*Formica lemani* might be hard to distinguish from its congeners, *F fusca* and *F. gagates*, hence the identification made by SC was confirmed via multivariate analyses of numeric traits using a modern numeric morphology-based alpha taxonomic key provided by Seifert [23]. The pubescence on the first gastral tergite (sqPDG) is very dense, scores vary between 3.46 and 3.87, which is in the lowest range of the *F. gagates* (3.6 to 10.8) and the highest scores of *F. lemani* and *F fusca* (2.4 to 3.5), but the dull cuticular surface of the workers and highly dense pubescence rule out the possibility of *F gagates*. The relatively high number of unilateral pronotal setae (4 to 15, nest sample mean 7.67) and the discriminant function provided by Seifert [23] place the nest sample in *F. lemani* with a very high certainty. The D5 scores vary between 6.03 and 8.62 (nest sample mean 7.15). Morphometric character recording and analyses were done by SC.

*F. polyctena*Förster, 1850: [52]

*F. pratensis*Retzius, 1783: [26]

        = *Formica congerens* Nylander, 1846: [25]

        = *Formica rufa pratensis*: [40]

*F. pressilabris*Nylander, 1846: [43]

*F. rufa*Linnaeus, 1761: [26]

*F. rufibarbis*Fabricius, 1793: [26]

        = *Formica cunicularia* Latreille, 1798: [26]

        = *Formica fusca* var. *glebaria* Nylander, 1846: [27]

        = *Formica fusca glebaria* Nylander, 1846: [67]

*F. sanguinea*Latreille, 1798: [25]

*F. truncorum*Fabricius, 1804: [27]

        = *Formica truncicola* Nylander, 1846: [25]

##### Genus: *Polyergus*

*P. rufescens* (Latreille, 1798): [25]

#### 3.5.3. Tribe: Lasiini

##### Genus: *Lasius*

*L. alienus* (Förster, 1850): [26]

        = *Formica aliena* Förster, 1850: [42]

*L. balcanicus*Seifert, 1988: [38]

*L. bicornis* (Förster, 1850): [27]

*L. bombycina* (Seifert and Galkowski, 2016) based on current findings of Seifert and Galkowski [30] *Lasius bombycina* is an eastern European congener of *L. paralienus* that has long been considered to occur in Hungary. The formerly believed continuous population of “*L. paralienus*” is therefore split into two geographically distinct species and the Hungarian population belongs to *L. bombycina*.

        = *Lasius paralienus* Seifert, 1992: [38]

*L. brunneus* (Latreille, 1798): [26]

*L. carniolicus*Mayr, 1861: [41]

*L. citrinus*Emery, 1922: [69]

        = *Lasius bicornis* var. *affinis* Schenck, 1852: [70]

        = *Lasius affinis* Schenck, 1852: [27]

*L. distinguendus* (Emery, 1916): [54]

*L. emarginatus* (Olivier, 1792): [26]

*L. flavus* (Fabricius, 1782): [26]

        = *Formica flava* Fabricius, 1782: [25]

*L. fuliginosus* (Latreille, 1798): [26]

        = *Formica fuliginosa* Latreille, 1798: [42]

*L. jensi*Seifert, 1982: [54]

*L. meridionalis *(Bondroit, 1920): [38]

*L. mixtus* (Nylander, 1846): [26]

*L. myops*Forel, 1894: [28]

        = *Lasius flavus* var. *myops* Forel, 1894: [27]

*L. neglectus* Van Loon, Boomsma et Andrásfalvy, 1990: [71]

*L. niger* (Linnaeus, 1758): [26]

        = *Formica nigra* Linnaeus, 1758: [42]

*L. nitidigaster* Seifert, 1996: [29]

*L. platythorax*Seifert, 1991: [38]

        = *Formica brunnea* Latreille, 1798: [25]

        = *Formica timida* Förster, 1950: [25]

*L. psammophilus*Seifert, 1992: [38]

*L. umbratus* (Nylander, 1846): [26]

        = *Formica umbrata* Nylander, 1846: [25]

##### Genus: *Prenolepis*

*P. nitens* (Mayr, 1853): [28]

        = *Tapinoma nitens*: [25]

        = *Prenolepis imparis* var. *nitens*: [26]

#### 3.5.4. Tribe: Plagiolepidini

##### Genus: *Plagiolepis*

*P. ampeloni* (Faber, 1969): [24]

*P. pallescens*Forel, 1889: [24]

*P. pygmaea* (Latreille, 1798): [26]

        = *Tapinoma pygmaeum*: [42]

*P. taurica*Santschi, 1920: [62]

        = *Plagiolepis vindobonensis* Lomnicki, 1925: [27]

*P. xene*Staercke, 1936: [41]

### 3.6. Species of Doubtful Occurrence

#### 3.6.1. *Temnothorax rabaudi* (Bondroit, 1918)

This species was reported from Hungary by Barrett [72] based on a male individual (in combination with *Leptothorax*), but it must have been a misidentification. The identification of *Temnothorax* species based only on males is possible in exceptional cases, hence such determinations should be considered uncertain. Moreover, *T. rabaudi* is a West European species, which may not stretch its distribution to the Carpathian Basin. Since neither confirmed information is available, nor has the voucher specimen been deposited to any public collection, this taxon is eliminated from the Hungarian checklist.

#### 3.6.2. *Tetramorium impurum* (Förster, 1850)

This species was identified by Csősz et al. [54] based on workers and sexual forms, but the samples definitely belong to *Tetramorium staerckei* Kratochvíl 1944. *Tetramorium impurum* is a mountain species and is unlikely to occur in the Carpathian Basin. Therefore this taxon is eliminated from the current checklist.

## 4. Discussion

Checklists are more than just simple lists of taxa. Inventorying what we have forms the solid basis of any nature conservation policy, but this information also serves as an indispensable tool for scientific field experiments. Ant taxonomy is a highly dynamic field, where new taxa are described or old names are revised even in the otherwise seemingly well-known European fauna every year [32,73], thus there is a need for a periodical update of any checklist, as is the case of the Hungarian myrmecofauna.

The Hungarian fauna and flora are characterized by a mixture of Eastern European, Central European and Mediterranean elements; many species having here their westernmost, easternmost or even northernmost location of their range of distribution. Additionally, there are quite a number of endemic animal and plant species, characteristic to the Pannonian biogeographical region, which harbors higher biodiversity compared to surrounding biogeographical regions, being mostly restricted to Hungary [74,75,76]. While in ants there are no endemic species or even subspecies known to Hungary, the Hungarian myrmecofauna is no exception to the above-mentioned characteristics. Submediterranean elements as *Catagplyhis*, *Cardiocondyla* and *Bothriomyrmex* species are present, along with typical European temperate climate species, as *Myrmica* and *Formica* s. str. species.

Since the publication of the last Hungarian checklist [24] several taxonomic novelties appeared starting from the thorough revision of the genus *Lasius* [30,77] to the clarification of the taxonomy of the extremely problematic genus *Tetramorium* [31,48], which is also very well reflected by the high species richness of both genera in the Hungarian myrmecofauna. Characteristic to the Hungarian myrmecofauna is the presence of some truly rare species, as *Lasius carniolicus* or parasitic *Plagiolepis* species. Moreover, there is quite a considerable number of indoor species as well confirming the fact that constant efforts have been put into the survey of the myrmecofauna in the past decades.

**Figure 1:**
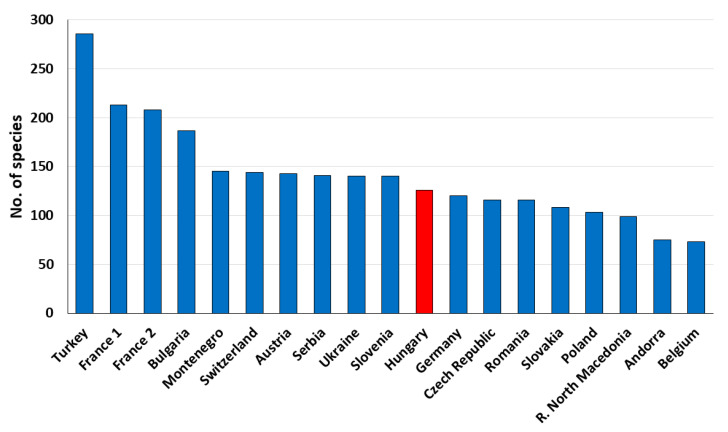
The number of known ant species in other European countries (Turkey: [17]; France 1: [20]; France 2: [19]; Bulgaria: [78]; Montenegro: [21]; Switzerland: [23]; Austria: [23]; Serbia: [79]; Ukraine: [14]; Slovenia: [80]; Germany: [23]; Romania: [13,16,32,81]; Czech Republic: [82]; Slovakia: [83]; Poland: [15]; Republic of North Macedonia: [84]; Andorra: [18]; Belgium: [85]).

In order to reveal the characteristics of any fauna, it should be put in the context of data known from neighboring countries (Figure 1). The biogeographic specificity of Hungary is also revealed by the high number of species compared to other countries in temperate Europe relative to the country’s size and geographical characteristics (Figure 1). Surprisingly, the number of Hungarian species is comparable to the myrmecofauna of the much more diverse Slovenia or Ukraine, while it surpasses the known number of species in Germany or Romania, which are larger countries with habitats ranging from high mountains to seasides. Certainly, the smaller number of known ant species in other countries could also be attributed to the relative lack of data as is, probably, the case of Romania (see [13] for arguments). However, such comparisons, and the fact that in the last decade only six new species were recorded, entitle us to say that the Hungarian myrmecofauna is quite well-known; many more new native species are not expected to occur, maybe with the exception of some parasitic ones with cryptic lifestyle. While the number of known species may not vary much in the forthcoming years, knowledge on the geographic distribution of Hungarian ant species is still incomplete. Thus, the implementation of further faunistic studies should be encouraged to complete the picture with the missing biogeographic data.

## 5. Conclusions

We provide a detailed list of the Hungarian ant fauna by adding the new elements found in the last decade including the annotated synonymic list of taxa.

Inventorying what we have forms the solid basis of any nature conservation policy. Such lists can also help to cope with recent challenges posed by agricultural intensification and climate change generated biodiversity crisis and serve indispensable information for scientific field experiments.

Awareness of fauna composition is a must to assess changes in our environment. The Hungarian fauna and flora are characterized by a mixture of Eastern European, Central European and Mediterranean elements; many species having here their westernmost, easternmost or even northernmost location of their range of distribution. Thereby our fauna can be considered one of the indicator areas that are affected by climatic changes.

In order to better highlight the characteristics of the Hungarian ant fauna, it is put in the context of data known from neighboring countries (Figure 1). The biogeographic specificity of Hungary is also revealed by the relatively high number of species compared to other countries in temperate Europe (Figure 1). Surprisingly, the number of Hungarian species is comparable to the myrmecofauna of the much more diverse Slovenia or Ukraine, while it surpasses the known number of species in Germany or Romania, which are larger countries with habitats ranging from high mountains to seasides.

These comparisons make us say that the Hungarian myrmecofauna is quite well-known, and many more new native species are not expected to occur. However, some cryptic parasitic ones or invasive elements can be expected to show up in the near future.

## Data Availability

No new data were created or analyzed in this study. Data sharing is not applicable to this article.
